# Development of a reconditioning program for elderly abdominal surgery patients: the Elder-friendly Approaches to the Surgical Environment–BEdside reconditioning for Functional ImprovemenTs (EASE-BE FIT) pilot study

**DOI:** 10.1186/s13017-018-0180-7

**Published:** 2018-05-21

**Authors:** Alyssa McComb, Lindsey M. Warkentin, Margaret L. McNeely, Rachel G. Khadaroo

**Affiliations:** 1 Faculty of Rehabilitation Medicine, University of Alberta, 1-38 Corbett Hall, 8205-114 St NW Edmonton, Edmonton, Alberta T6G 2G4 Canada; 2 Department of Surgery, University of Alberta, Edmonton, Alberta Canada; 3 Department of Critical Care Medicine, University of Alberta, Edmonton, Alberta Canada

**Keywords:** Reconditioning, Rehabilitation, Elderly, Abdominal, Surgery, Emergency, Physiotherapy, Bed rest

## Abstract

**Background:**

Elderly individuals who are hospitalized due to emergency abdominal surgery spend over 80% of their recovery time in bed, resulting in early and rapid muscle loss. As these elderly individuals have a lower physiological reserve, the impact of muscle wasting on function may be profound. The objectives of this study are to (1) create an independently led post-surgical reconditioning program and (2) pilot its implementation, while assessing the feasibility and safety of the program.

**Methods:**

The BE FIT program was generated with hospital rehabilitation staff to target lower limb strength, balance, and endurance. This pilot study was assessed using a sequential before and after trial, with a cohort of patients aged ≥ 65 years enrolled in the Elder-friendly Approaches to the Surgical Environment (EASE) study. Change in 30-s sit-to-stand performance between postoperative day 2 and discharge was compared between Usual Care pre- and post-BE FIT participants.

**Results:**

A total of 66 patients participated in the sub-study, 33 Usual Care and 33 BE FIT. Mean (SD) age was 76.2 (8.78); 44 (67%) were female, with 11 (17%) reporting mild/moderate frailty on the CHSA Clinical Frailty Scale. BE FIT participants had a median of three rehab days and self-reported completing an average of 83% of the exercises. The adjusted between group difference showed that the BE FIT patients were able to complete more stands than the Usual Care (1.9 stands (0.94), *p* = 0.05). There were no reported adverse events.

**Conclusion:**

The reconditioning program was shown to be safe and feasible within the hospital setting for the elderly emergency abdominal surgery patients. More rigorous assessment is needed to confirm this effectiveness and to better assess patient adherence to self-directed exercise.

**Trial registration:**

Registration #NCT02233153 through ClinicalTrials.gov. Registered September 8, 2014.

## Background

With our rapidly aging population and advancements to allow surgery as a viable option for those with increased age, a greater number of surgical procedures are now performed on seniors (≥ 65 years) in Canada and the USA [1–3]. Unfortunately, the increased prevalence of pre-existing vulnerabilities and the precipitating insults associated with surgery (polypharmacy, catheters, and bed rest) result in higher rates of adverse events in the elderly population compared to their younger counterparts [4, 5]. Occurrence of these adverse events often leads to longer hospital stays, more intensive care, greater resource expenditure, and an increased risk of mortality [4–9].

The traditional approach to postoperative surgical care has largely centralized around activity restrictions to optimize wound healing. However, prolonged bed rest is not a viable option for the frail elderly as evidence strongly supports its detrimental effects on muscle mass that can compromise independence [10]. Currently, there is no consensus on the necessary level or type of activities that should be restricted following surgery and current physical therapy practices focus on mobilization and discharge practices, as opposed to enhancing muscular strength [10].

The American College of Surgeons National Surgical Quality Improvement Program (ACS-NSQIP) Best Practices Guideline suggests early mobilization with a focus on positioning assistance, balance, and gait training [11]. Newer enhanced recovery after surgery care pathways encourage early mobilization with the suggestions that patients dangle their legs on the day of surgery, then ambulate every 4 to 6 h each day while they are awake until discharge beginning on postoperative day 1 [12]; however, even in well-established enhanced recovery programs, mobilization adherence is low [13], with seniors having lower adherence than non-senior adults [14]. Adult patients often do not sit upright until postoperative day 1 and take an average of 5.3 days to independently mobilize [15]; previous research by Browning et al. [15] reported total median uptime was 3.0 (IQR 8.2) min for the first postoperative day, increasing to only 4.4 (IQR 65.6) min on the fourth postoperative day.

For these reasons, new rehabilitation strategies are needed to improve functional outcomes in older adults following abdominal surgery, especially for those done urgently when pre-admission optimization is not feasible [16]. The objective of this pilot was to develop and test an exercise program that considered patient healing and safety, promoted self-management, and was resource neutral in terms of the workload impact on hospital staff.

## Methods

### Development of the BE FIT rehabilitation program

Prior to program development, the author AM, a kinesiologist, shadowed in-hospital physical and occupational therapists who were responsible for the standard physical therapy of the population group of interest. The purpose of this was to familiarize the author with current hospital rehabilitation practices, safe movement with recent incision sites, common areas of physical weakness for elderly inpatients, and the accessibility of the hospital ward. The program was based around the progressive use of multi-disciplinary teams within Alberta’s Health Care system. The goal of the program was to provide a uniform transitional program that all members of the health-care team would be knowledgeable about and comfortable using. The program was designed with input from exercise physiologists, physical therapists, occupational therapists, and surgeons.

An initial list of exercises was selected by authors AM and MM, based on expert opinion and the American College of Sports Medicine’s recommendations for elderly individuals [17], with the guiding intention that the exercises were simple enough that a lay person could teach, be taught, or perform the exercises with minimal guidance. These exercises targeted lower limb strength, balance, and endurance and would be expected to support participants in independent living and ambulation once discharged. The exercises chosen were reviewed by all authors, as well as the in-hospital physical and occupational therapists, and consensus was reached for all exercises included in the final program, after several rounds of edits and discussion. Three exercise levels, of increasing difficulty, of the program were generated, and visual instructional materials and patient logbooks were created (Table 1). The program materials were trialed with non-study participant elderly surgical inpatients prior to implementation for user feedback and refinement.

**Table 1:** BE FIT reconditioning program exercises

CHSA Clinical Frailty Scale [19]	BE FIT exercises
Very fit/well	Standing-based program • Sit-to-stand • Calf raises • Hip marching
Managing well/vulnerable	Chair-based program • Bed rolls • Sit-to-stand (with assist) • Leg extensions
Mild/moderately frail	Bed-based program • Quad extensions • Butt squeezes • Triceps extensions

Participants were instructed to complete each exercise 10 times, thrice daily

### Pilot feasibility and safety assessment: study design

Testing of the BEdside reconditioning for Functional ImprovemenTs (BE FIT) program was done using a prospective, sequential before-and-after sub-study of the Elder-friendly Approaches to the Surgical Environment (EASE) study [18]. The main EASE study’s aim is to assess the impact of an elder-friendly surgical unit through capacity alignment, an interdisciplinary care team, an evidence-informed care pathway, the BE FIT reconditioning program, and transition optimization. The full protocol of the EASE study is published, and the Human Research Ethics Boards of the University of Alberta granted ethics approval (Pro00047180) [18].

Participants for EASE-BE FIT sub-study were consecutively enrolled elderly patients (≥ 65 years old) who had been referred directly to Acute Care Surgery services for emergency abdominal surgery at the University of Alberta Hospital in Edmonton, Canada, from May 2015 to May 2016. Patients were excluded if they (1) received elective or palliative surgery, (2) were admitted due to trauma, (3) were dependent in three or more activities of daily living or were non-ambulatory prior to admission, and (4) could not read/speak or comprehend English. The research coordinator approached the eligible patients prior to their surgery, and willing participants were consented.

Participants included prior to the implementation of the BE FIT program received only usual care, which may have included physical therapy if it was requested by the clinical team (Usual Care cohort). The physiotherapist would assess the frequency and timing of the clinical physiotherapy involvement, independent of the study. Participants included after the BE FIT program implementation received usual care plus the BE FIT reconditioning program beginning on postoperative day 2 (BE FIT cohort). The appropriate level of the program was determined using the participant’s frailty within 2 weeks prior to admission, assessed using CHSA Clinical Frailty Scale [19], along with informal interview of the participant on postoperative day 2. The exercises were demonstrated to the participant, and the first set of exercises was supervised to ensure they were being performed safely. The participants were instructed to perform the exercises three times per day until discharged, and the visual diagram of the exercises was provided to remind the participant of proper technique. The logbook was provided to encourage compliance, and the participants were told to notify the clinical or study team if they needed re-teaching of the exercises or modifications; however, as the program was intended to be self-directed, the study team did not initiate follow-up with the participant nor monitor adherence to the exercises.

### Data collection

Chart review was completed to determine age, sex, race, marital status, living situation prior to admission, type of surgery, postoperative physical and occupational therapy intervention, any occurrence of in-hospital falls, discharge status, and length of stay.

Participants completed a 30-s sit-to-stand (STS) test on postoperative day 2 (prior to receiving the BE FIT program for the BE FIT cohort) and again on the day of discharge. The STS test is a measurement used to assess functional lower extremity strength in older adults [20]. Briefly, a participant rises to a full stand from being seated in a chair, without the use of their arms, and then returns back to the initial seated position. The number of full repetitions that are completed in 30 s is recorded. As all participants had recent abdominal incisions, participants were permitted to use their arms to assist them into a standing position if they felt necessary. More repetitions are indicative of better functional performance.

Six weeks after discharge, for those participants who returned for their scheduled follow-up with their surgeon, a Timed Up and Go (TUG) test was completed [21]. The TUG test is a commonly used screening tool for falls risk. The participant is timed while they rise from a chair, walk 3 m at a comfortable and safe pace, turn and walk back to the chair, and sit down again. Faster time indicates a better functional performance.

### Sample size

Sample size was calculated based on the few normative values currently published in scientific literature, along with the established minimal clinically important difference scores (2.6 stands in the STS [22] and 3.5 s in the TUG [23]). For a statistical significance of *p* = 0.05 and a power value of 0.90, the necessary value for the STS tests was based on the mean and standard deviation values normalized by Jones et al. [24] on community-dwelling older adults, suggesting a minimum sample of 11. For the TUG, using mean and standard deviation scores normalized by Steffen et al. [25], a sample size of 22 is suggested. We therefore attempted to enroll a minimum of 60 participants (30 in each group) to increase power and attempt to have equivalent distribution of baseline characteristics between groups.

### Analyses

#### Change in functional status

Participants were included in the analysis regardless of their length of stay or adherence to the program. Descriptive statistics were calculated and compared between Usual Care and BE FIT groups using the *t* test/Mann-Whitney test for continuous variables and chi-squared/Fisher exact test for categorical. Change in STS was calculated by subtracting postoperative day 2 STS from the discharge STS within participants. Change in STS and post-discharge TUG test results were compared between Usual Care and BE FIT groups first by using a two-sample *t* test, then in multivariable linear regression models in order to adjust for sex, CHSA Clinical Frailty Scale, and surgery type.

#### Adherence and informal feedback

Adherence was calculated by averaging number of times they self-reported completing the reconditioning program a day (maximum 3) by the number of possible rehab days (discharge date minus postoperative day 2 date). Informal feedback and participant comments about the program were captured on the logbook and notes written by the research coordinator during final testing at discharge.

## Results

### Participant characteristics

A total of 66 participants were enrolled in the study with 33 participants in each group. The Usual Care group had more intestinal revision or resection surgeries (*p* = 0.001), had fewer cholecystectomies (*p* = 0.04), were more often mild or moderately frail (*p* = 0.02), and received more physical or occupational therapy intervention (*p* = 0.009) compared to the BE FIT group (see Table 2). Four participants in the BE FIT group did not perform the reconditioning program, as they were discharged on postoperative day 2; for those who completed the program (*n* = 29), median number of possible rehab days was 3 (IQR 1–6) and mean (SD) adherence was reported as 2.56 (0.65), suggesting that participants completed 83% of their exercises. The logbook was completed by 18 (55%) of BE FIT participants. Two falls occurred during the duration of the study (one Usual Care, one BE FIT), but neither was associated with performance of the functional testing or completion of the BE FIT program.

**Table 2:** Participant characteristics

Characteristics	Usual Care	BE FIT	*P* value
	*n* = 33	*n* = 33	
Age (years)*	77.3 (9.56)	75.1 (7.92)	0.31
Female	22 (67)	22 (67)	1.0
White	31 (94)	29 (88)	0.22
Married	19 (58)	17 (52)	0.621
CHSA Clinical Frailty Scale			0.07
Very fit/well	9 (27)	12 (36)	
Managing well/vulnerable	15 (45)	19 (58)	
Mild/moderately frail	9 (27)	2 (6)	
Procedure			0.02
Appendectomy	1 (3)	2 (6)	
Cholecystectomy	4 (12)	11 (33)	
Hernia repair	3 (9)	5 (15)	
Intestine resection/revision	25 (76)	12 (36)	
Other	0 (0)	3 (9)	
PT/OT visit	16 (48)	6 (33)	0.009
Falls	1 (3)	1 (3)	1.0
Length of stay (days)^†^	9 (7–13)	6 (3–11)	0.02

Reported as *n* (%) unless indicated otherwise

*Mean (SD)

^†^Median (IQR)

### Participant feedback on program

Several BE FIT participants responded with comments such as “More exercises,” “Enjoyed the program, wish I could take it home,” and “Please provide this to other hospitals” in the logbook and commented that it was helpful to be given direction on moving after surgery, as they felt unsure if it was allowed. However, four (12%) of participants felt that postoperative day 2 was too soon to complete exercises after surgery (either due to pain or beliefs that bed rest would be most beneficial to them) and either delayed beginning the program or chose not to complete the exercises during their hospital stay. No concerns were brought forth by the clinical team regarding the program.

### Sit to stand

**Fig. 1:**
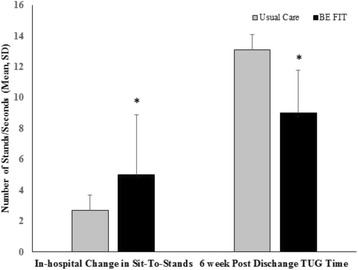
Functional status outcomes pre- and post-BE FIT. TUG, Timed Up and Go. *p < 0.05 compared to Usual Care, after adjusting for sex, CHSA Clinical Frailty Scale, and surgery type

Overall, 10 (15%) of participants completed the STS reps without using their arms, 37 (56%) completed their STS reps with the assistance of using their arms, and 19 (29%) used a combination of without-arm and with-arm reps. Combining all reps, regardless of arm use, the BE FIT group completed 5.0 (3.9) more stands at discharge compared to postoperative day 2, while the Usual Care group completed 2.7 (3.0) (between group difference 2.3 (0.45) stands, *p* = 0.01). After adjusting for sex, Clinical Frailty Scale, and surgery type, the difference of difference was 1.9 (0.94) (*p* = 0.05) (see Fig. 1).

### Timed Up and Go

Loss to follow-up at 6 weeks post-discharged occurred for 19 (56%) participants in the Usual Care group and 10 (30%) participants in the BE FIT group. BE FIT participants completed the TUG in 9.0 (2.8) s, while the Usual Care completed it in 13.1 (5.2) s (difference between groups, 4.0 (1.3), *p* = 0.004). After adjusting for sex, Clinical Frailty Scale, and surgery type, the difference between groups remained significant (mean difference 4.1 (1.4), *p* = 0.006) (see Fig. 1).

## Discussion

This is one of the first pilot studies to develop a structured postoperative reconditioning program for elderly emergency surgery patients. Using an integrated knowledge translation approach to generating the BE FIT program contributed to the successful implementation for the program. The reconditioning program was feasible and safe within our hospital setting. There were no reported associated adverse events, and the self-directed nature of the program and use of a progress monitoring via logbook allowed us to remain resource neutral. Both the change in STS between postoperative day 2 and discharge and the TUG at 6 weeks post-discharged showed greater improvement in the BE FIT group, compared to Usual Care, even after adjusting for sex, clinical frailty, and surgery type. Leaving the hospital in a better functional state places BE FIT participants to a reduced risk of injury and falls and loss of physical independence [6].

As there are few studies of this nature available in the literature, similar results are limited; however, improvement in STS scores due to early exercise interventions has been found in patients undergoing elective hip and knee arthroplasty [26], elderly acute general medical patients [27, 28], sedentary community-dwelling older adults [29], and nursing home residents [30].

Most participants were eager and willing to perform the exercises as often as they felt they could; however, a small portion of participants felt that they should not be performing exercises so soon after surgery and therefore did not complete the program. Self-reported adherence was adequate, though overall use of the logbook was variable. While this was not formally assessed, we expect barriers to logbook use likely include time constraints, misunderstanding around logbook use, or sensory impairments (example, eye glasses for reading unavailable at bedside). Unfortunately, participants who did not perform the program also did not complete the logbook, creating reporting bias and limiting our knowledge of the barriers to exercise in this group. Participants’ informal feedback regarding exercise after surgery prompts us to believe that the lack of education and sharing information around postoperative mobility exists at the level of not only heath care providers but patients and their families as well. Education opportunities for health-care staff may increase uptake of postoperative mobility programs and should be assessed and incorporated into further research.

Unfortunately, the concurrent EASE interventions, small sample size, and unclear adherence due to poor completion rates of logbooks limit our interpretation of this sub-study. For these reasons, a dedicated study is required to determine the optimal program and resources necessary to improve patient outcomes. Our future work will be to perform a pragmatic randomized controlled trial examining our exercise intervention, tailored to frailty.

## Conclusions

As this sub-study included a small cohort and occurred in the setting of the concurrent implementation of other elder-friendly initiatives, our ability to interpret our finding and report unequivocal success is limited. However, our pilot reconditioning program results show promise in helping offset declines in physical function in elderly patients following emergency abdominal surgery and warrants further, more rigorous investigation to assess patient uptake and adherence, safety, and effectiveness.

## Abbreviations

BE FIT: BEdside reconditioning for Functional ImprovemenTs
EASE: Elder-Friendly Approaches to the Surgical Environment
STS: Sit-to-stand
TUG: Timed Up and Go
